# Informational and support needs of patients with head and neck cancer: current status and emerging issues

**DOI:** 10.1186/s41199-016-0017-6

**Published:** 2016-11-11

**Authors:** Carolyn Y. Fang, Carolyn J. Heckman

**Affiliations:** Cancer Prevention and Control Program, Fox Chase Cancer Center, 333 Cottman Ave, Philadelphia, PA 19111 USA

**Keywords:** Informational needs, Treatment side effects, Communication, Caregivers, Health behaviors, HPV

## Abstract

The objective of this article is to review and summarize the extant literature on head and neck cancer (HNC) patients’ informational needs and to characterize emerging issues in this patient population in order to define priorities for future research. HNC patients may undergo challenging treatment regimens and experience treatment-related alterations in primary daily functions such as speech and eating. These changes often persist following treatment and may lead to significant deficits in quality of life and interpersonal relations. Despite empirical evidence demonstrating that receipt of adequate information and support is predictive of improved outcomes post-treatment, relatively limited attention has been paid to the informational and support needs of HNC patients. This review focuses primarily on three topic domains: (1) managing treatment-related side effects; (2) addressing alcohol and tobacco dependence; and (3) informational needs in the areas of human papillomavirus (HPV) and clinical trials. While there is increasing awareness of the rehabilitation and survivorship needs in this patient population, patients note that the impact of treatment on social activities and interactions is under-discussed and of key concern. In addition, there is a significant gap in addressing communication and informational needs of caregivers and family members who are integral for promoting healthy behaviors and self-care post-treatment. Greater integration of programs that address tobacco or alcohol dependency within a comprehensive treatment and support plan may increase patient motivation to seek help and enhance patient success in maintaining long-term abstinence. Finally, emerging patient-provider communication needs, particularly in the context of decision making about clinical trials or surrounding an HPV-related diagnosis, have been noted among both patients and healthcare providers. Future research on the development of novel programs that offer feasible and acceptable methods for addressing unmet informational and support needs is warranted and may yield benefit for improving patient-reported outcomes.

## Background

Despite advances in diagnostic tools and treatment modalities, treatment for head and neck squamous cell carcinoma (HNC) can lead to considerable long-term functional impairment. Patients may experience difficulty swallowing, dry mouth, nutritional deficits, pain, and declines in social functioning, speech, and sexuality [1]. Physical and psychosocial complaints can persist even following successful therapy [2], and patients often report significant decrements in quality of life (QOL), poorer interpersonal relations, and increased social isolation [3].

Notably, empirical data indicate that patients’ perceptions of having obtained adequate information and support are predictive of positive rehabilitation outcomes in the 2- to 6-year post-treatment period [4]. However, despite the complexity of treatment regimens and challenging recovery process, relatively limited attention has been paid to the informational and support needs of HNC patients. Therefore, the objective of this article is to briefly review and summarize the extant literature on informational and support needs and to characterize emerging issues in this patient population to help define priorities for future research.

## Main text

### Symptom management and treatment side effects

HNC patients undergoing surgical treatment may experience physical alterations on a part of their body that is highly visible (i.e. face) and with significant implications for social interactions. As a result, several studies focused on issues of postoperative changes in appearance or speech [5–7]. In a cross-sectional study of 280 surgical patients who were surveyed regarding body image [5], 75 % reported feeling concerned or embarrassed by bodily changes related to the cancer and/or its treatment at some point following diagnosis. The concerns most commonly expressed by patients involved scarring and disfigurement (42.3 %), speaking-related issues (35.8 %), loss of teeth (30.1 %), and social eating (25.1 %) [5]. Other concerns included swelling, drooling, hair loss, and weight loss. And despite patients’ overall levels of satisfaction with their healthcare team, a subgroup of patients had unmet informational needs. Specifically, 25 % of patients reported dissatisfaction with the information received about the degree of scarring/disfigurement to be expected following surgery; 32 % were dissatisfied with information received about the potential effects of radiation treatment on physical appearance, and 44 % reported dissatisfaction with information about potential effects of chemotherapy on physical appearance. Factors associated with dissatisfaction included age and time since diagnosis, with younger patients reporting higher levels of dissatisfaction and body image concerns. Levels of dissatisfaction were also found to increase after surgery and remain elevated one-year post-surgery. Overall, more than one-third of patients reported that greater informational resources to help manage multiple concerns would have been helpful [5].

Among surgical patients, a desire for greater pre-operative education and preparation for post-operative changes has been reported [8], particularly in the domains of speech [9] and eating [8, 10]. In an early study, questionnaires assessing informational needs and support services were completed by 125 patients who had undergone laryngectomy for treatment of their cancer and 28 spouses. Among patients, 21 % were not satisfied with the information received prior to surgery and were unaware preoperatively of the inability to speak after surgery [9]. Importantly, 61 % of patients and 81 % of spouses reported that the information provided was insufficient [9]. A smaller study evaluated informational needs and eating-related experiences among 34 patients who were recruited from an Internet-based laryngectomy support group. Similar to prior findings, most patients reported dissatisfaction with the information received, particularly with regard to the effects of laryngectomy on loss of taste and changes in eating [10]. Importantly, the majority of patients and spouses desired to have a conversation about how treatment may impact social activities and interactions with friends, family members, and other individuals in a public setting [8].

Of note, studies have revealed a potential disconnect between what doctors believe their patients are worried about and what patients say they are worried about. Healthcare professionals perceived that speech impairment and self-image were patients’ primary concerns after surgery; however, patients rated concerns about the physical consequences of treatment and its interference with social activities as key concerns post-surgery [11]. Alterations in the social nature of eating are frequently reported by patients as topics that deserve more attention [8, 10]. In addition, patients desire more information on nutrition and strategies for coping with loss of smell or taste [10]. In the absence of receiving appropriate referrals to nutritionists or other support professionals, patients often turn to internet Web sites or support groups to seek the information they desire [10, 12], although the information obtained from these sources may not be reliable or accurate. While healthcare professionals may wish to avoid burdening the patient with detailed nutrition and dietary recommendations during the early stage of rehabilitation, patients do not perceive advice to “just eat something” to be particularly helpful [10], further underscoring the disconnect between patients and their providers.

In addition to swallowing and speech issues, there is growing awareness that HNC patients experience sleep disturbance. Among 58 HNC patients who completed a cross-sectional survey on sleep and fatigue, greater sleep dysfunction was associated with younger age and higher symptom burden [13]. Multiple factors may contribute to sleep dysfunction and poor sleep quality, as observed in a longitudinal, prospective study of 457 HNC patients in which pain, xerostomia, depression, presence of a tracheotomy tube, and younger age were significant predictors of poor sleep quality one-year post-diagnosis [14]. However, treatment type (surgery, radiation, or chemotherapy), primary tumor site, and cancer stage were not significantly associated with sleep quality over time.

Sleep disruption may be due to not only pain or symptom burden, but also the physiologic alterations resulting from the tumor or cancer treatment. Both radiation and surgical treatments lead to changes in the upper airway that can contribute to the development of obstructive sleep apnea (OSA) [15]. Although there have been few studies of OSA in HNC patients, the limited data available suggest that HNC patients are at increased risk of OSA [16–19]. One study of 17 HNC patients who were assessed prior to surgical treatment revealed the presence of OSA in 13 patients (76 %) [18]. A retrospective review of 56 HNC patients who had also undergone formal sleep evaluation found that patients with active disease or who had received radiation therapy were more likely to have OSA [20]. And in contrast to the general population, where obesity is a risk factor for the development of OSA, HNC patients with OSA are not more likely to be obese or overweight [15, 20, 21], suggesting that anatomic changes resulting from the tumor or cancer treatment may predispose patients to the development of OSA. Because many symptoms of OSA are nonspecific (daytime sleepiness, insomnia, dry mouth upon awakening) and could be attributed to other factors, patients may be unaware that they are suffering from a potentially treatable sleep disorder [22]. Clinicians and healthcare providers can help patients identify symptoms of OSA and provide referrals to sleep specialists if warranted.

Other unmet informational and support needs that have been identified pertain to financial and psychosocial domains. In a prospective study of 82 newly diagnosed HNC patients, informational needs commonly reported at pre-treatment included issues involving financial support (78 %), access to support groups for patient or partner (52 %), treatment side effects (50 %), and how treatment may impact QOL and functioning over the next year (43 %) [23]. Patients were hetereogenous with respect to treatments received (surgery only, radiation only, or a combination of surgery, radiation, and chemotherapy) and were re-assessed 1 month post-treatment, at which time 60 % of patients continued to report that they had not received any information regarding financial support, 34 % had not received information about support groups, and 28 % had not received information about treatment side effects or how treatment may impact functioning over time [23]. In light of the fact that study respondents had access to healthcare and were recruited from research hospital settings (which may have more resources to offer patients), it is possible that these data are underestimates of financial and other concerns than what might be found in the broader HNC patient population. To date, the informational and support needs of HNC patients who are uninsured, lack access to healthcare, or are being managed in community practices or other settings is not well-characterized.

Family caregivers often play an important role in supporting HNC patients and providing assistance during and after treatment [24, 25]. Similar to patients, caregivers of HNC patients report many unmet informational needs [24, 26]. Among 59 family caregivers of HNC patients who had completed radiation therapy, nearly 75 % reported high informational needs at diagnosis [24]. Although caregivers’ needs decreased over time, over half of caregivers still had high informational needs at the end of treatment. Predominant concerns included reducing patient pain and distress across the treatment trajectory. Similarly, in a qualitative study of oral cancer patients and their family caregivers, caregivers requested more information on pain management and self-care [26]. Finally, both patients and caregivers desired more information regarding managing side effects and nutrition issues [26].

The extant literature has characterized patients’ unmet informational and support needs across multiple domains, but limitations of the existing research include small sample sizes in many studies, wide variability in the timing of study assessments relative to treatment and across studies, and a reliance on predominantly cross-sectional study designs. Most studies utilized a convenience sample comprised of predominantly non-Hispanic whites, and therefore informational and support needs of HNC patients from other racial/ethnic subgroups are not well-described. In addition, the studies conducted have tended to use varied measures to assess informational needs and satisfaction with information received, thereby making it difficult to compare across studies. However, a standardized measure of satisfaction with information has been developed and evaluated specifically among HNC patients [27]. This measure, the Satisfaction with Cancer Information Profile (SCIP), assesses patient satisfaction with the amount and content of information, as well as the form and timing of the information received [27]. The use of standardized and validated measures will help promote greater consistency in the assessment and identification of informational needs over time across diverse HNC patient subgroups.

Multimedia interventions can offer a promising approach to addressing communication and information needs. In one study, partners of HNC patients received a multimedia-based program including a patient booklet, interactive computer booth and take-home DVD, which was delivered by a nurse practitioner [28]. Partners of HNC patients who received the intervention reported higher levels of satisfaction with the information provided compared to control group participants; importantly, intervention participants had lower levels of anxiety and depression at 3–6 months [28]. Other Internet-based programs that deliver content and features tailored to one’s role (cancer survivor or caregiver) have been found to be well-received by oral cancer survivors and their family caregivers; and they offer the potential added ability to use online tools for communicating with other survivors and caregivers [26].

Due to the diversity of individual needs and preferences, tailored communication materials may represent the best strategy for addressing informational gaps [29, 30]. As might be expected, younger HNC patients (29–49 years of age) were more likely to report having Internet access compared to older patients (65+ years), and data suggest varied preferences regarding the format in which information is delivered [12], with some patients preferring to receive information delivered in-person by a healthcare specialist [30], whereas others are receptive to Internet-based programs [26]. Higher education level and female gender were also associated with greater preference for Internet-based programs [12]. Finally, patient financial concerns and access to healthcare services remain a significant area of informational need that is not well-addressed and one that warrants greater attention. In view of the complexity of treatment-related side effects and the extensive rehabilitation that may need to occur both during and post-treatment, strategies to address patients’ informational and support needs across the continuum of care (from pre-treatment through post-treatment) are critically needed.

### Alcohol and tobacco dependence

Following a cancer diagnosis and/or treatment, patients may seek to maintain or improve their health via alterations in lifestyle behaviors such as physical activity, diet, smoking, or alcohol use. Given that cancer treatment and subsequent side effects can adversely affect physical functioning and nutritional status, numerous studies have focused on the effects of physical activity or nutritional support interventions on patient outcomes (please see comprehensive reviews on physical activity [31–33] and nutrition [34–36] in HNC patients). In contrast, there have been relatively fewer studies designed to address alcohol or tobacco dependency for those patients with addiction disorders. Empirical data suggest that alcohol abuse and untreated alcohol withdrawal syndrome can lead to treatment complications and poorer outcomes, including failure of extubation or ventilator weaning and higher rates of mortality [37, 38]; however, there has been limited research focused on delivering specialized services to treat comorbid alcoholism among HNC patients [39, 40]. One US study published in 2010 found that 44.5 % of HNC survivors reported current alcohol use 1 year after diagnosis [41]. Several studies have reported various factors to be associated with ongoing alcohol use after diagnosis including: male gender, early-stage disease, longer time since treatment, receiving chemotherapy and/or radiation therapy compared to surgery, and superior oral functioning [41–43]. A 2016 Spanish study reported that alcohol use decreased by 16.7 % after diagnosis, and that receiving a surgeon’s recommendation about alcohol was strongly associated with alcohol reduction or cessation [44]. Only one randomized controlled trial published in 2006 by Duffy and colleagues tested an alcohol intervention for HNC patients. Patients with HNC and tobacco, alcohol, or depression disorders, about half of whom were veterans, were randomized to a nurse-administered cognitive-behavioral and pharmacotherapy intervention for treatment of tobacco, alcohol, and depression [39]. No significant differences in 6-month alcohol consumption were observed between the intervention condition and usual care [39], attesting to the challenges of addressing alcohol disorders in this patient population.

There is a relatively larger body of research on tobacco-related complications. Continued smoking after the diagnosis of HNC impacts treatment and risk for complications, increases the likelihood of recurrence and second primary cancers, and adversely affects QOL and survival [45–48]. According to a 2015 systematic review of six studies, the survival rate for HNC patients who continued smoking after diagnosis was 21–35 % lower than patients who quit, and the recurrence rate for active smokers was 23–30 % higher than for quitters [47]. In a matched-pair study of HNC and nicotine-dependent patients published in 2007, HNC patients who stopped using tobacco also had lower rates of tobacco-related second primary tumors [46].

HNC patients, like other smokers, demonstrate ambivalence about quitting, and smoking rates among HNC patients have remained relatively constant over the last two decades despite the availability of an increased number of empirically-based pharmacotherapy and behavioral interventions to treat tobacco dependence [49]. Many HNC patients who are smokers are interested in quitting, and may even succeed in quitting unassisted, at least initially. However, a significant minority of smokers are unable to quit even after diagnosis of a smoking-related cancer, and initial quitters are at high risk for relapse. Only Duffy and colleagues’ randomized controlled trial published in 2006 found a significant treatment effect of a nurse-delivered cognitive-behavioral and pharmacotherapy tobacco cessation intervention compared to usual care for HNC patients [39].

Despite the fact that evidence-based tobacco cessation interventions are available, they are woefully underutilized and have been minimally evaluated among HNC patients. Providers have not fully implemented the Public Health Service 5A guidelines for tobacco cessation (ask, advise, assess, assist, arrange) [50] among patients with tobacco-related cancers – this is likely due to a multitude of barriers that include patient factors (e.g., low motivation, self-efficacy, knowledge), provider factors (e.g., limited training, self-efficacy, time), and system factors (e.g., high cost, minimal insurance coverage). Two studies of lung and HNC patients combined demonstrated partial implementation of the 5A’s, as assessed by self report and medical chart review, with 93 % of patients reporting that they were asked about tobacco use, 76–86 % advised about tobacco use, 46 % assessed for their interest in quitting, 40–65 % assisted, but only 4 % arranged with follow-up [49, 51]. A study published in 2011 found that individualized cessation programs were preferred by patients, and younger patients with early stage disease and those with partners who smoked were more interested in cessation programs [25]. A 2012 study found that delivery of 5A’s was most likely to occur when patients requested cessation advice from their providers. However, according to a small qualitative study conducted at a large NCI-designated comprehensive cancer center in the Southeast published in 2009, patients do not ask providers for help quitting or maintaining abstinence, and relapsed patients are hesitant to disclose smoking due to feelings of stigma and guilt [28]. In addition, clinicians vary in the types of assistance they provide and their awareness and sensitivity to relapsed patients' concerns [28]. The advice and type of assistance offered by providers may not resonate with patients—specifically, providers reported emphasizing the long-term risks of continued smoking in their interactions with patients, but lung and HNC patients expressed a preference for a balance between risks of smoking and benefits of quitting/abstinence [52].

Few empirically-supported programs targeted for or tested specifically with HNC patients exist. The results of three randomized trials for HNC patients have been published. In the first study published in 1993, the intervention consisted of surgeon- or dentist-delivered advice to stop smoking, a contracted quit date, tailored written materials, and booster advice sessions compared to a usual care advice control condition [53]. At randomization, 88 % were current smokers. At 12-month follow-up, 70 % of participants completing the trial were continuous abstainers; among baseline smokers alone, the continuous abstinence rate was 65 %. However, the intervention effect was not statistically significant. Predictors of continuous abstinence were surgery rather than radiation only, greater stage of change, younger age, lower nicotine dependence, and race/ethnicity other than white, non-Hispanic. Based on these findings, the authors recommended systematic brief advice to stop smoking for HNC patients, with a stepped-care approach for patients less able to quit [53].

In Duffy and colleagues’ study published in 2006, Veterans Affairs Medical Center (VAMC) patients with HNC and tobacco, alcohol, or depression disorders were randomized to a nurse-administered cognitive-behavioral and pharmacotherapy intervention for treatment of tobacco, alcohol, and depression [39]. Significant differences in 6-month smoking cessation rates were found with 47 % quitting in the intervention condition compared to 31 % in the usual care condition [39]. However, a recent randomized study of VAMC HNC patients published in 2016 found that receiving a $150 monetary incentive at each test visit (1, 3, and 6 months) for being biochemically-confirmed abstinent based on urine cotinine testing -- in addition to being paid $50 to attend each of three cessation classes provided by the VAMC -- did not result in improved cessation rates [54].

Despite the mixed results that have been reported to date, it is imperative that providers take advantage of the “teachable moment” of a diagnosis of HNC to advise smokers to quit, which can be quite effective [50]. Tobacco use and cessation should be addressed on an ongoing basis with patients throughout treatment and survivorship, including among recent quitters in order to prevent relapse. Too often, a sporadic “Band-Aid” approach is used for tobacco cessation, even among cancer patients, despite calls for nicotine dependence to be managed as a chronic disease [55, 56]. In 2015, the National Comprehensive Cancer Network published guidelines for tobacco cessation intervention among cancer patients [57], but the successful implementation of these guidelines remains to be evaluated.

### Emerging issues

#### Human papillomavirus

A topic not commonly addressed is the impact of cancer and its treatment on sexual functioning [1, 58, 59]. In an early study, HNC patients who had extensive facial disfigurement following surgical treatment reported worse relations with their partners, increased social isolation, and reduced sexuality compared to patients with minor disfigurement [6]. Due to significant improvements in facial reconstruction techniques, extensive disfigurement is now less common; but sexual functioning may still be impacted due to treatment-related effects on breathing, swallowing, and speaking. Alterations in any of these functions can have a negative impact upon intimacy, communication, or other sexual activities. Indeed, although 85 % of HNC patients reported moderate-to-high interest in sexual relations, a majority (58 %) reported arousal problems, and 58 % did not engage in sexual intercourse [59]. Further, 51 % considered the quality of their current sexual functioning to be poor, with younger patients (<65 years) reporting significantly poorer sexual functioning than patients aged 65 or older [59].

In addition, data suggest that the incidence of human papillomavirus (HPV)-associated head and neck cancers is increasing [60–62]. Accumulating evidence point to HPV as a causal factor in the etiology of head and neck cancers that arise among non-smokers and non-drinkers [63, 64]. This emerging subgroup of HNC patients has specific informational needs, and many clinicians find themselves ill-prepared for these discussions. Patients may have questions about HPV-related disease that are difficult to answer due to limited data or physician discomfort with discussing matters related to sexual health [65].

Among HNC patients receiving an HPV-related diagnosis, informational and psychosocial needs are particularly apparent. In a qualitative study of 10 men with HPV-related HNC, the majority (8/10) sought additional HPV-specific information, mostly from the Internet [66]. Patient knowledge about HPV varied significantly, and many had questions regarding transmission of HPV infection and potential consequences for current or future partners [66]. Among 62 HNC patients who had a long-term partner, only two-thirds of patients correctly reported that they had an HPV+ tumor [67]; the remainder of patients were unsure or reported that they did not have an HPV+ tumor. Only a minority of patients (35 %) reported that their cancer was caused by HPV [67]. The majority of patients reported being uninformed about what precautions (if any) needed to be taken to prevent HPV transmission to their partner, and 39 % reported that their oncologist did not discuss issues related to HPV with them. Over half (58 %) reported seeking additional information from sources other than their oncologist [67].

The sexually-transmitted nature of HPV may create challenges for healthcare professionals who have limited experience discussing sexual health with their patients. In a qualitative study of 15 healthcare professionals who treat patients with oropharyngeal cancers, providers described two primary challenges: (1) discomfort or lack of familiarity with talking about sexual health and behaviors; and (2) limitations of scientific knowledge about the virus [68]. Patients may desire answers to specific questions about how they became infected with HPV, when they became infected, who the infection was transmitted from, and the likelihood of re-infection after cancer treatment – questions that are difficult to answer [68]. These data highlight significant challenges and gaps in patient-provider communication with respect to HPV-related issues and point to the need for additional research in this area. At present, existing data on the informational needs of patients with HPV-related cancers are quite limited as the few studies that have been conducted are based on relatively small samples of patients and physicians recruited from large academic research institutions, which may not be representative of the broader patient and healthcare provider population. As a result, little is known about the informational and support needs of patients being managed in community practices and their community providers. Studies designed to foster the development of valid and comprehensive educational and support programs to address HPV-related issues would be extremely beneficial for both patients and their providers.

#### Decision making about clinical trials

Another area of emerging need is the provision of informational support for clinical trials. While clinical trials are essential to the evaluation of new therapeutic regimens, a relatively small proportion of cancer patients participate in these trials [69]. This is likewise true for clinical trials specifically recruiting HNC patients, where it was reported that over 25 % of HNC trials that had been terminated, suspended, or withdrawn was due to insufficient accrual of patients [70]. Two studies investigating barriers to recruitment of HNC patients were identified. In one study, 85 healthcare professionals involved in clinical trial research (investigators, research nurses) completed a web-based survey on barriers to clinical trial recruitment. The most commonly reported barriers to recruitment (identified by more than 50 % of respondents) included patient refusal to consent due to treatment preference or aversion to randomization, the complexity and amount of information that needs to be provided to patients, and lack of time to support research [71], which is consistent with studies in other cancer patient populations [72]. Further exacerbating this situation is the complexity of the information that needs to be accurately conveyed, which can be particularly challenging for patients with limited health literacy [73].

Hence, the utilization of decision aids to assist with informed decision making about clinical trial participation has been explored as a potentially useful tool to help patients identify their values and goals, consistent with making an informed choice. To date, there have been no decision aids developed to specifically support HNC patients’ informational needs and decision making about clinical trials. But prospective, randomized studies involving mixed cancer patient populations have yielded promising results. For example, among 1255 cancer patients who were randomly assigned to receive either a web-based program that provided tailored, interactive educational content about clinical trials (intervention condition) or general written information about clinical trials (control condition), intervention participants reported significantly greater increases in knowledge and greater decreases in attitudinal barriers compared to control participants [74]. Thus, tailored educational programs can effectively deliver key information about clinical trials and may help enhance communication about and preparation for decision making about clinical trials. Future research is needed to evaluate whether similar programs yield comparable benefits in addressing HNC patients’ informational needs and reducing barriers to clinical trials.

## Conclusion

Across studies, many patients desire additional information and support, particularly with respect to managing treatment-related side effects, maintaining one’s health and healthy lifestyle behaviors after treatment, and understanding an HPV-related diagnosis. Despite the growing body of research on rehabilitation and survivorship needs in this patient population, patients note that the impact of treatment on social activities and interactions is under-discussed and of key concern. Interventions focused on swallowing and improving jaw mobility (for a review see [75]) often neglect to address the psychological and/or social aspects of eating and drinking, issues reported to be of high importance among survivors and which can contribute to decrements in emotional and social well-being. In addition, there is a significant gap in addressing communication and informational needs of caregivers and family members who provide considerable levels of support to the patient and are integral for promoting healthy behaviors and self-care during and after treatment. As caregivers often express different concerns than patients do, future studies that address caregivers’ specific needs are warranted.

In addition, there is a need for more fully integrated programs to provide support for managing substance dependency issues. For example, many HNC patients express interest in quitting smoking and attempt to quit, but fewer follow through with enrolling in evidence-based smoking cessation programs or are successful in maintaining long-term abstinence. Motivational interventions to facilitate enrollment into formal programs that address alcohol or tobacco dependence among cancer patients may be beneficial [76]. Studies suggest that a cancer diagnosis and subsequent treatment window offers a “teachable moment” during which patients may be motivated to initiate and maintain healthy behaviors, including smoking cessation and decreased alcohol use [77]. Ultimately, programs addressing tobacco or alcohol dependency that are incorporated into a comprehensive treatment plan may decrease the stigma associated with substance abuse and increase patient motivation to seek help and support for staying healthy after treatment.

Finally, two emerging areas of informational needs that warrant greater attention include: (1) communication about an HPV-related diagnosis and its impact on intimacy; and (2) support for decision making about clinical trials. Although patients with HPV-related disease desire more information regarding HPV and head and neck cancer, communication and practical barriers (such as physician time constraints, limited knowledge, and patient or physician discomfort in discussing sexual health) reduce patient satisfaction with the information provided. With the increasing prevalence of HPV-related HNCs, corresponding programs that address patient and partner concerns regarding HPV-related issues are greatly needed. Similarly, multiple challenges exist in the enrollment of HNC patients to clinical trials, including limited time for conveying large amounts of complex information, addressing informational needs of patients and family members, and discussing patient preferences and values. Findings derived from other cancer patient populations suggest that novel web-based programs may not only be an effective and cost-efficient approach for delivering such information, but may also represent an acceptable and feasible format for communicating information about multiple topics that can be tailored to meet the unique needs of patients and family members.

## Abbreviations

HNC: Head and neck cancer
HPV: Human papillomavirus
VAMC: Veterans Affairs Medical Center
